# STATE PERFORMANCE ON HOME- AND COMMUNITY-BASED SERVICES EXPENDITURES UNDER THE BALANCING INCENTIVES PROGRAM

**DOI:** 10.1093/geroni/igz038.2951

**Published:** 2019-11-08

**Authors:** Regina A Shih, Esther M Friedman, Bonnie Ghosh-Dastidar, Jose Escarce

**Affiliations:** 1 RAND, Arlington, Virginia, United States; 2 RAND Corporation, Santa Monica, California, United States; 3 RAND, Santa Monica, California, United States; 4 UCLA, Los Angeles, California, United States

## Abstract

Medicaid has been at the forefront of policy efforts to “rebalance” long-term services and supports (LTSS) from institutional toward home- and community-based services (HCBS). The Balancing Incentives Program (BIP), authorized under the Affordable Care Act, sought to increase spending on HCBS relative to total LTSS among states that were spending less than 50% of total Medicaid LTSS dollars on HCBS each year. BIP’s benchmark for states to spend more than 50% of LTSS on HCBS was meant to improve states’ infrastructure to ensure an equitable, consumer-friendly process to access HCBS. For the first time, we quantified how states performed on HCBS expenditures. We analyzed state-level HCBS expenditure data for states eligible for BIP (n=35) from 2008 to 2015, which includes several years both before and after BIP implementation. We examined the effect of BIP on HCBS expenditures as a percent of total LTSS spending using fixed-effects modeling which controls for stable characteristics of states and years. We find that BIP states increased HCBS spending relative to non-BIP states, and this effect continued to grow over our study period. In the first year after BIP implementation, BIP states increased spending by 1.8% relative to non-BIP states. The difference in spending between BIP and non-BIP states is even larger in subsequent years (4.8% in year 2, 5.3% in year 3, and 5.8% in year 4). Results indicate that BIP prompted increased state-level spending that continued to rise several years after initial implementation of BIP.

